# A Room Temperature Trimethylamine Gas Sensor Based on Electrospinned Molybdenum Oxide Nanofibers/Ti_3_C_2_T_x_ MXene Heterojunction

**DOI:** 10.3390/nano14060537

**Published:** 2024-03-18

**Authors:** Shiteng Ma, Jingyu Guo, Hao Zhang, Xingyan Shao, Dongzhi Zhang

**Affiliations:** College of Control Science and Engineering, China University of Petroleum (East China), Qingdao 266580, China; mashiteng89@163.com (S.M.); guojingyu1997@126.com (J.G.); zhshy2020zhh@163.com (H.Z.); shao2053850672@163.com (X.S.)

**Keywords:** electrospinning, MoO_3_ nanofibers, Ti_3_C_2_T_x_ MXene, trimethylamine sensor, p-n heterojunction

## Abstract

The combination of two-dimensional material MXene and one-dimensional metal oxide semiconductor can improve the carrier transmission rate, which can effectively improve sensing performance. We prepared a trimethylamine gas sensor based on MoO_3_ nanofibers and layered Ti_3_C_2_T_x_ MXene. Using electrospinning and chemical etching methods, one-dimensional MoO_3_ nanofibers and two-dimensional Ti_3_C_2_T_x_ MXene nanosheets were prepared, respectively, and the composites were characterized via XPS, SEM, and TEM. The Ti_3_C_2_T_x_ MXene–MoO_3_ composite material exhibits excellent room-temperature response characteristics to trimethylamine gas, showing high response (up to four for 2 ppm trimethylamine gas) and rapid response–recovery time (10 s/7 s). Further, we have studied the possible sensitivity mechanism of the sensor. The Ti_3_C_2_T_x_ MXene–MoO_3_ composite material has a larger specific surface area and more abundant active sites, combined with p–n heterojunction, which effectively improves the sensitivity of the sensor. Because of its low detection limit and high stability, it has the potential to be applied in the detection system of trimethylamine as a biomarker in exhaled air.

## 1. Introduction

With the rapid development of science and technology and the improvement of people’s living needs, the requirement for high-performance sensors has become increasingly urgent [1]. Trimethylamine (TMA) is a common toxic gas that causes serious damage to the eyes, nose, and skin. In addition, prolonged exposure may seriously affect the nervous system [2]. Therefore, it is very important to prepare new sensitive materials and develop new preparation technology to improve the high-performance detection of TMA. In daily life, TMA can be utilized as a biomarker to evaluate the freshness of fish and other seafood. Moreover, TMA is often used to assess the degree of spoilage in seafood. This is because, after the death of the seafood, the concentration of TMA will increase correspondingly with the breakdown of trimethyl-N-oxide. When the concentration of TMA exceeds 10 ppm, it is generally regarded as being rotten [3]. In addition, some patients with certain diseases, such as trimethylaminuria, are unable to metabolize trimethylamine, leading to its elimination from the body through sweat, urine, and exhaled breath [4]. Therefore, detecting the concentration of TMA in exhaled breath can serve as a diagnostic method for diseases such as renal disorders and trimethylaminuria. Zhang et al. developed a MoO_3_/V_2_O_5_ TMA gas sensor for disease detection using a hydrothermal synthesis method. This sensor has the potential for rapid detection of TMA at room temperature, showing great promise in non-invasive disease diagnosis [5]. Therefore, TMA concentration detection is an important index in seafood deterioration evaluation and human health monitoring, and it is necessary to develop a high-performance TMA sensor.

In previous studies, metal oxide semiconductor (MOS) materials have been extensively utilized in the construction of gas sensors, such as SnO_2_, In_2_O_3_, Fe_2_O_3_, and Co_3_O_4_, owing to their advantages like low production cost, fast response, miniaturization, and good reliability [6,7,8,9]. Molybdenum trioxide (MoO_3_) is a typical N-type semiconductor gas-sensitive material, and its surface contains a multitude of acidic sites that can react with basic TMA, which makes it an important material for TMA detection [10]. For example, Shen et al. synthesized MoO_3_ nanosheets using the solvothermal method. The porous nanosheet TMA sensor has more oxygen vacancies, so the response value to 50 ppm TMA at a lower operating temperature (133 °C) is as high as 198 [11]. Zhou et al. synthesized MoSe_2_/MoO_3_ composite material using a two-step hydrothermal method, which exhibited excellent sensing performance towards TMA gas. The response/recovery time reached the sub-second level, with a low detection limit down to the ppb range [12]. Yan et al. designed a porous, hollow Co_3_O_4_@ZnO structure for the TMA sensor using a simple ZIF-_67_@ZIF_8_-derived method. The sensor shows good TMA resistance sensing performance with high sensitivity and fast response/recovery time. The sensor also has better baseline stability [13].

In recent years, numerous scholars have conducted in-depth research on the applications of two-dimensional nanomaterials such as graphene [14], molybdenum disulfide (MoS_2_) [15], and tin selenide (SnSe_2_) [16] in the field of sensors. Their characteristics of high specific surface area and high surface activity make them extremely important functional materials in the field of sensor-sensitive materials [17,18]. MXene is an emerging two-dimensional transition metal carbide or nitride composed of transition metal nitride, carbide, and carbonitride. Its unique properties have attracted much attention in the field of nanotechnology, and it has been used to distinguish various inorganic gases and volatile organic compounds through chemical etching Ti_3_AlC_2_ [19,20,21]. Ti_3_C_2_T_x_ is one of the most extensively studied MXenes, which possesses a high specific surface area and conductivity and is capable of adsorbing numerous functional groups on its surface [22,23]. Ti_3_C_2_T_x_ MXene provides a matrix with unique gas adsorption capacity and good electrical conductivity, enabling heterojunction-based room-temperature sensing [24]. With these unique advantages, Ti_3_C_2_T_x_ MXene is widely used for gas detection at room temperature. He et al. successfully synthesized MXene modified using SnO_2_ nanoparticles via a hydrothermal method. The formed MXene/SnO_2_-based heterojunction sensor has excellent sensitivity to different NH_3_ concentrations from 0.5 to 100 ppm at room temperature [24]. Zhang et al. prepared a high-performance trace ammonia sensor based on (001) TiO_2_/MXene. Using the synergistic effect of TiO_2_ and MXene (001) crystal surface, the sensing performance of the sensor is effectively enhanced, and the ultralow detection limit of 156 ppt is achieved, which can conveniently and economically detect food deterioration [25]. The MXene and SnO_2_ have different Fermi levels that can drive the charge transfer at the heterojunction interface, enrich the electrons on the surface area of SnO_2_, and improve the sensitivity of the Ti_3_C_2_T_x_ MXene–SnO_2_-based gas sensor.

In this study, Ti_3_C_2_T_x_ MXene–MoO_3_ composite material was prepared using electrospinning and chemical etching methods, forming a p–n heterojunction. The Ti_3_C_2_T_x_ MXene–MoO_3_ composite material was characterized using SEM, XPS, XRD, and other methods. The sensing performance of the sensor was thoroughly investigated, and the sensor exhibited excellent sensitivity, rapid response, outstanding selectivity, and good long-term stability to TMA. Through the analysis of the sensing mechanism of the sensor, it was further demonstrated that heterojunction could effectively enhance gas sensing performance. In addition, a TMA alarm detection system was constructed based on the Ti_3_C_2_T_x_ MXene–MoO_3_ sensor, achieving rapid alarm for TMA exceeding the limit. This study provides a theoretical basis and solutions for the development of non-invasive disease detection and food spoilage detection.

## 2. Experimental

### 2.1. Materials

The ammonium heptamolybdate [(NH_4_)_6_Mo_7_O_24_·4H_2_O, 99%, CAS: 12054-85-2], polyvinylpyrrolidone (PVP, Mw = 1,300,000, CAS: 9003-39-8), hydrochloric acid (HCl, analytical grade, CAS: 7647-01-0), titanium aluminum carbide powder (Ti_3_AlC_2,_ CAS: 196506-01-1), anhydrous ethanol (CH_3_CH_2_OH, 99.7%, CAS: 64-17-5), N,N-dimethylformamide (DMF, 99.5%, CAS: 68-12-2), and lithium fluoride (LiF, 99%, CAS: 7789-24-4) used in this study were all purchased from National Pharmaceutical Chemistry Reagents.

### 2.2. Material Synthesis

The MoO_3_ nanofibers were prepared using a combination of electrospinning and calcination. Firstly, DMF and anhydrous ethanol were mixed in a ratio of 1:1. Then, 0.8 g of PVP and 0.3 g of ammonium heptamolybdate were dissolved in 20 mL of the mixed solvent, and the solution was stirred magnetically at 60 °C for 12 h. When the solution became transparent, the electrospinning precursor was obtained. Subsequently, the prepared precursor was moved to a syringe (No. 21) and subjected to a voltage of 16 kV (feeding rate of 0.707 mL/h and distance of 15 cm) to obtain ammonium heptamolybdate/PVP nanofibers. Finally, the composite nanofibers were calcined at 500 °C for 2 h to completely remove the organic polymer components.

**Figure 1:**
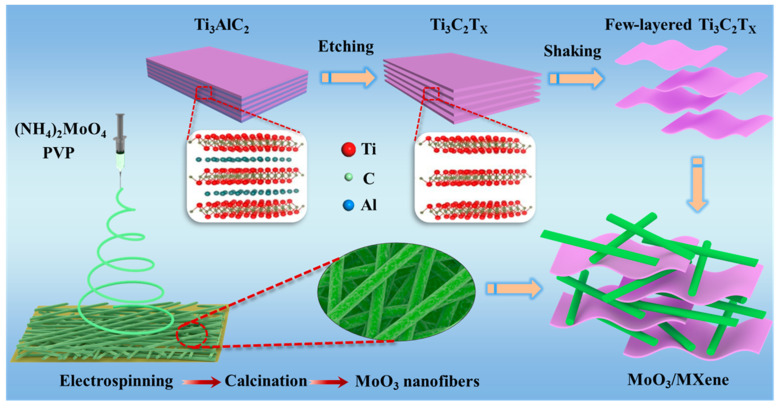
The preparation process of the Ti_3_C_2_T_x_ MXene–MoO_3_ nanocomposite.

Delaminated Ti_3_C_2_T_x_ MXene with great dispersion in water was synthesized through etching Ti_3_AlC_2_ [19], as shown in Figure 1. The MoO_3_ nanofibers and Ti_3_C_2_T_x_ MXene solution were dissolved in ethanol with magnetic stirring, and the Ti_3_C_2_T_x_ MXene–MoO_3_ mixture suspension was obtained for subsequent use.

### 2.3. Test Device

The Ti_3_C_2_T_x_ MXene–MoO_3_ hybrid suspension was assembled on the electrode surface using a layer-by-layer coating technique [26,27,28]. The material was fully and evenly dissolved in volatile ethanol, ground with a mortar for 20 min, and then subjected to ultrasonicated to disperse the sample more evenly in ethanol. Finally, the surface of the sensor is coated with composite sensing materials layer by layer using a brush and dried for aging so that the Ti_3_C_2_T_x_ MXene–MoO_3_ sensor is prepared.

**Figure 2:**
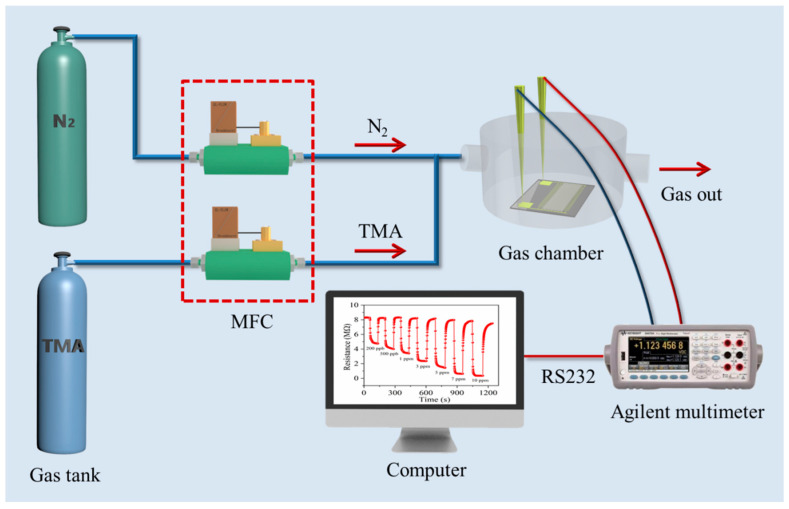
Schematic illustration of the TMA-sensing measurement system.

The TMA test system is shown in Figure 2, which mainly includes TMA, N_2_, gas flow controller, test chamber, Ti_3_C_2_T_x_ MXene–MoO_3_ sensor, data logger, and host computer. A data logger (Agilent 34970A) is used to acquire the Ti_3_C_2_T_x_ MXene–MoO_3_ sensor response. The sensor was tested in a 750 mL closed gas chamber. A certain volume of standard TMA gas is extracted with a syringe and mixed with pure air to obtain different concentrations (0.2–10 ppm) of TMA gas. Based on the real-time monitoring of the Agilent 34970a, the change in the resistance of the sensor under TMA gas is recorded, and the test results are displayed on the computer. During the test, the sensor is first placed in an N_2_ environment to obtain a stable fundamental resistance.

During the testing phase of the sensor, its response (S) is defined as ǀR_a_ − R_g_ǀ/R_a_ × 100%, where R_g_ and R_a_ are the resistance values of the sensor before and after the introduction of the target gas molecule, respectively. The sensor response and recovery time are the time it takes for its resistance value to reach 90% of the total resistance variation range.

### 2.4. Characterization

The samples were imaged using a field emission scanning electron microscope (Hitachi SU8020, the equipment is manufactured by Hitachi High-Tech Co., Ltd. in Tokyo, Japan) and a transmission electron microscope (TEM, JEM-2100, the equipment is manufactured by Hitachi High-Tech Co, Ltd., Tokyo, Japan). MXene, MoO_3_, and Ti_3_C_2_T_x_ MXene–MoO_3_ composites were characterized using a Bruker D8 ADVANCE (The device is manufactured by Bruker Corporation, Billerica, MA, USA) phase X-ray diffractometer (XRD). X-ray photoelectron spectroscopy (XPS) was conducted using a Thermo escalab 250Xi scanning (TEC, USA, the equipment is manufactured by Thermo Fisher Scientific, Waltham, MA, USA).

## 3. Results and Discussion

### 3.1. Sample Characterization

**Figure 3:**
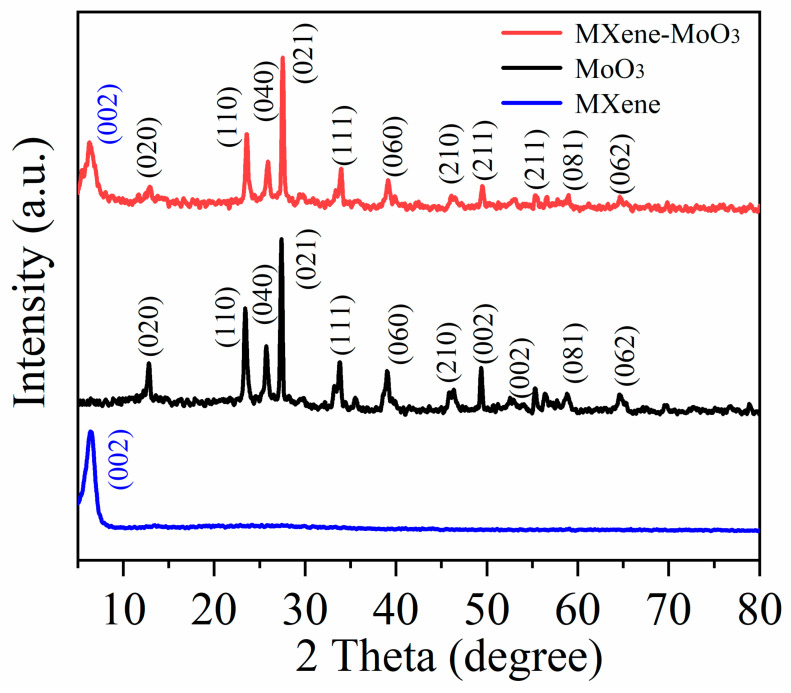
XRD pattern of the Ti_3_C_2_T_x_ MXene–MoO_3_ nanocomposite.

The XRD analysis of Ti_3_C_2_T_x_ MXene, MoO_3_, and Ti_3_C_2_T_x_ MXene–MoO_3_ nanocomposites was performed using an X-ray diffractometer (Rigaku D/Max 2500PC). As illustrated in Figure 3, the diffraction peaks observed in the acquired samples are effectively matched with the orthorhombic phase of MoO_3_ (JCPDS No.89-5108) and Ti_3_C_2_T_x_ MXene (JCPDS No.29-0095). The diffraction peaks of MoO_3_ are centered at 2θ = 13.2°, 23.8°, 26.1°, 27.4°, 38.6°, 42.3°, 46.6°, and 53.3°, corresponding to the (020), (110), (040), (021), (060), (141), (200), and (211) crystal planes, respectively [29]. During the calcination process, the complete elimination of PVP ensured the absence of any additional impurity peaks in the observed spectrum. Three high-intensity diffraction peaks can be observed, which indicates that MoO_3_ is a highly ordered orthorhombic crystal. The blue curve represents the widened peak observed at 7.7°, indicative of the (002) plane of Ti_3_C_2_T_x_ MXene, characterized by a lattice constant of 14.3 Å [30]. The X-ray diffraction (XRD) examination of the Ti_3_C_2_T_x_ MXene–MoO_3_ manifests congruent characteristic peaks corresponding to both MoO_3_ and Ti_3_C_2_T_x_ MXene, providing robust confirmation regarding the successful synthesis of the Ti_3_C_2_T_x_ MXene–MoO_3_ nanocomposite. The observed identical peaks in the XRD spectrum affirm the efficacious formation of the composite material, underscoring the precision and effectiveness of the synthesis process employed.

**Figure 4:**
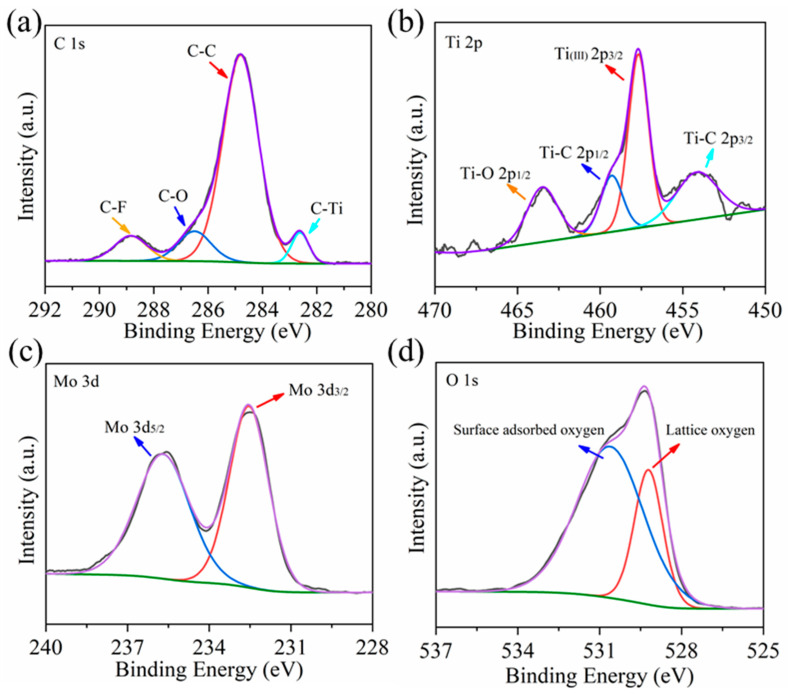
XPS spectra of Ti_3_C_2_T_x_ MXene–MoO_3_ sample: (**a**) C 1s core level spectrum, (**b**) Ti 2p core level spectrum, (**c**) Mo 3d core level spectrum, and (**d**) O 1s core level spectrum.

XPS characterization is used to determine the surface chemical composition and element valence of Ti_3_C_2_T_x_ MXene–MoO_3_. In the C 1s spectrum (Figure 4a), four distinctive fitting peaks are discernible at 282.6, 284.7, 286.6, and 288.9 eV, respectively, representing the C-Ti, C-C, C-O, and C-F bonds, thereby confirming the characteristic carbon bonding in Ti_3_C_2_T_x_ [31]. The Ti 2p spectrum exhibits four identifiable peaks, as anticipated in Figure 4b. The binding energies at 454.1, 457.7, 459.3, and 463.5 eV are attributed to Ti-C (2p_3/2_), Ti (II) (2p_3/2_), Ti-C (2p_1/2_), and Ti-O (2p_1/2_) bonds, respectively [32]. Figure 4c shows the Mo 3d energy level spectrum. The prominent symmetrical peaks, with a center at 235.6 eV and 232.5 eV, are indicative of Mo 3d_5/2_ and Mo 3d_3/2_ states within MoO_3_, respectively. The XPS spectrum of the O element, depicted in Figure 4d, illustrates a low binding energy peak at 529.2 eV, attributed to the Mo-O bond within MoO_3_, while a high binding energy peak at 530.7 eV signifies the presence of oxygen adsorbed on the sample’s surface [29].

**Figure 5:**
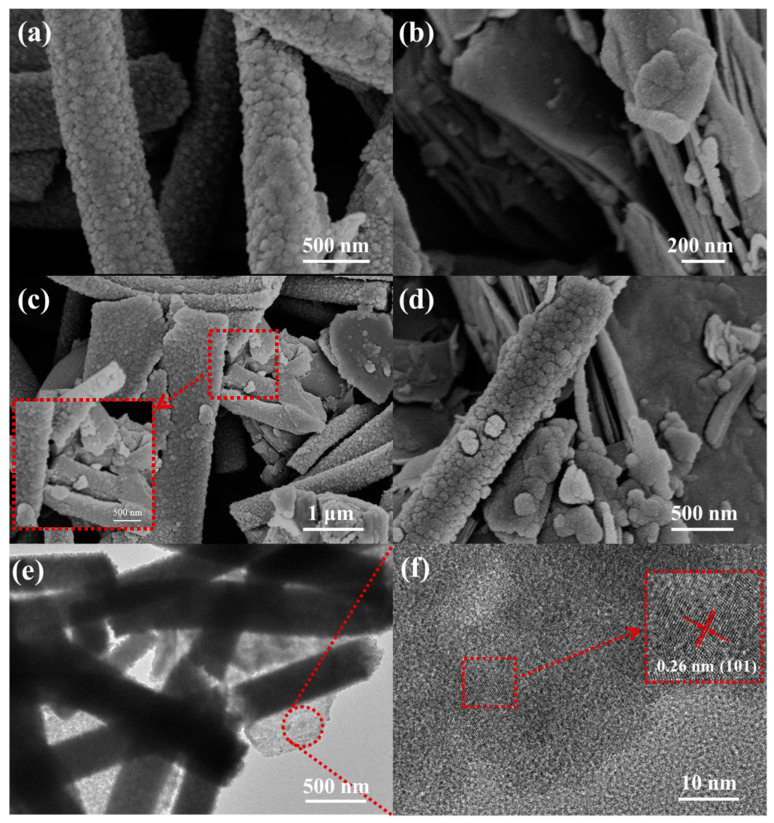
SEM images of (**a**) MoO_3_ nanofibers, (**b**) accordion-like MXene, and (**c**,**d**) Ti_3_C_2_T_x_ MXene–MoO_3_ nanocomposite. (**e**,**f**) TEM images of Ti_3_C_2_T_x_ MXene–MoO_3_ nanocomposite.

Figure 5 shows the scanning electron microscope and transmission electron microscope images of MoO_3_, Ti_3_C_2_T_x_ MXene, and Ti_3_C_2_T_x_ MXene–MoO_3_ heterostructure. Figure 5a is an image of MoO_3_ nanofibers. The diameter of the fibers is about 500 nm, and they are composed of closely arranged tiny particles. Figure 5b shows the typical accordion-like structure of Ti_3_C_2_T_x_ MXene. Figure 5c,d are the SEM images of the Ti_3_C_2_T_x_ MXene–MoO_3_ composite after ultrasonic treatment. It can be seen that the MoO_3_ nanofibers and the flake-like Ti_3_C_2_T_x_ MXene are tightly combined after the composite material is ultrasonically processed. Figure 5e,f show TEM images of Ti_3_C_2_T_x_ MXene–MoO_3_. From Figure 5e, we can observe the composite morphology of nanofibers with a diameter of 500 nm and Ti_3_C_2_T_x_ MXene. After magnifying the TEM image of Ti_3_C_2_T_x_ MXene, the existence of lattice fringes can be clearly observed. For example, the crystal spacing of 0.26 nm corresponds to the 101 crystal plane of Ti_3_C_2_T_x_ MXene. In conclusion, the SEM and TEM characterization images clearly demonstrate the effective synthesis of the Ti_3_C_2_T_x_ MXene–MoO_3_ heterostructure. The SEM and TEM analyses provide compelling visual evidence, showcasing the successful fabrication of the heterostructure.

### 3.2. TMA Sensing Properties

**Figure 6:**
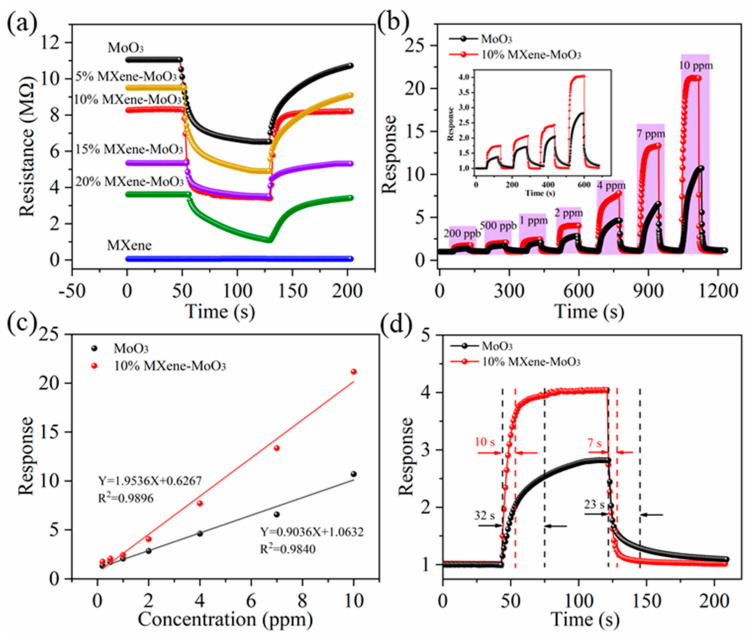
(**a**) The effect of different doping amounts of MXene on sensor performance under 1 ppm TMA gas. (**b**) Response curves of MoO_3_ sensor and 10% Ti_3_C_2_T_x_ MXene–MoO_3_ sensor at 200 ppb–10 ppm TMA gas concentration. (**c**) Fitting relationship between gas concentrations and sensor response. (**d**) Response and recovery characteristics of the sensor to 2 ppm TMA gas at 25 °C.

To comprehensively assess the gas sensing capabilities of the Ti_3_C_2_T_x_ MXene–MoO_3_ composite materials, a comprehensive array of measurements was undertaken to evaluate various parameters, including sensor sensitivity, stability, response time, and additional performance indicators. This thorough investigation involved detailed analyses aimed at elucidating the sensor’s effectiveness in detecting target gases, ensuring a comprehensive understanding of its operational characteristics. The entire testing process was conducted at room temperature (25 °C), with a relative humidity (RH) of 67% in the testing environment. First, we explored the effect of different masses of Ti_3_C_2_T_x_ MXene doping on sensor performance. As shown in Figure 6a, we measured the response of 0%, 5%, 10%, 15%, and 20% mass. When the sensor is exposed to TMA gas, the resistance will be significantly reduced. This phenomenon indicates that the Ti_3_C_2_T_x_ MXene–MoO_3_ composite exhibits typical n-type behavior. The data reveal that among the tested compositions, the 10% Ti_3_C_2_T_x_ MXene–MoO_3_ exhibits the most elevated response magnitude and demonstrates the swiftest response and recovery times. However, Ti_3_C_2_T_x_ MXene exhibits p-type behavior for TMA, and the response value is not high. This is because MXene acts as an electron donor rather than the main sensing material, so the appropriate MXene doping amount determines the final performance of the sensor. Then, we tested the performance of the 10% Ti_3_C_2_T_x_ MXene–MoO_3_ under different TMA gas concentrations. The response of the Ti_3_C_2_T_x_ MXene–MoO_3_ sensor was obtained by adjusting the concentration of TMA gas in the test chamber through the gas flow controller. The findings are depicted in the graphical representation presented in Figure 6b. The sensor demonstrates good response and recovery characteristics for different concentrations of TMA gas, with the detection limit as low as 0.2 ppm. In this work, the detection limit of the sensor is 0.2 ppm. Tests have been performed at concentrations below 0.2 ppm, and the results show that the sensor can detect lower concentrations but inconsistently. In keeping with scientific rigor, the detection of this sensor is limited to 0.2 ppm in this article. As the concentration of TMA gas increased from 0.2 ppm to 10 ppm, the response value of the 10% Ti_3_C_2_T_x_ MXene–MoO_3_ sensor increased from 1.74 to 21.2. In contrast, the response value of the pure MoO_3_ nanofiber gas sensor increased only from 1.33 to 10.7. It is worth noting that both the Ti_3_C_2_T_x_ MXene–MoO_3_ and MoO_3_ sensors exhibit excellent linear relationships between their response and gas concentration, as shown in Figure 6c. The linear fitting equations are Y = 1.9536X + 0.6267 (R^2^ = 0.9896) and Y = 0.9036X + 1.0632 (R^2^ = 0.9840). Furthermore, the incorporation of Ti_3_C_2_T_x_ MXene significantly enhances the response speed of the sensor, possibly due to its high specific surface area and surface activity. The response/recovery time of the 10% Ti_3_C_2_T_x_ MXene–MoO_3_ sensor to 2 ppm TMA was reduced to 10 s/7 s. Compared to the response/recovery time (32 s/23 s) of the MoO_3_ gas sensor, the MXene–MoO_3_ gas sensor exhibits a faster response/recovery speed in TMA gas sensing, as shown in Figure 6d. This may be attributed to the higher electron transfer efficiency and excellent conductivity of Ti_3_C_2_T_x_ MXene in gas-sensitive sensing nano-composite materials.

**Figure 7:**
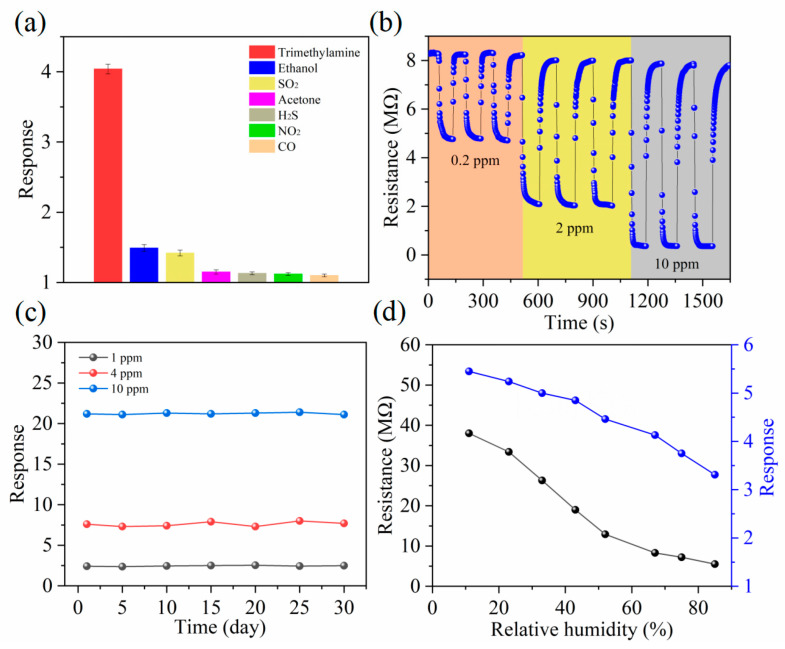
(**a**) Selectivity of the Ti_3_C_2_T_x_ MXene–MoO_3_ sensor toward 2 ppm of various gas species at 25 °C in 5 repeat experiments. (**b**) Repeatability of the Ti_3_C_2_T_x_ MXene–MoO_3_ sensor. (**c**) Long-term stability of the Ti_3_C_2_T_x_ MXene–MoO_3_ sensor toward 1, 4, and 10 ppm TMA gas at 25 °C. (**d**) Effect of relative humidity on the initial resistance and TMA-sensing properties of the Ti_3_C_2_T_x_ MXene–MoO_3_ sensor at 25 °C.

To assess the gas selectivity of the MXene–MoO_3_ sensor comprehensively, a diverse array of gases, such as hydrogen sulfide (H_2_S), ethanol, nitrogen dioxide (NO_2_), sulfur dioxide (SO_2_), and carbon monoxide (CO), were specifically chosen for testing purposes. This meticulous selection of gases aims to provide a thorough examination of the sensor’s ability to discern and respond to different gas types, thereby enhancing the depth of our understanding regarding its selective detection capabilities. When the gas concentration is uniformly 2 ppm, the 10% Ti_3_C_2_T_x_ MXene–MoO_3_ sensor exhibits a higher response to TMA compared to other gases, as shown in Figure 7a. This indicates that the MXene–MoO_3_ sensor possesses excellent selectivity. The sensor was placed at 0.2 ppm, 2 ppm, and 10 ppm TMA gas concentrations, and the resistance curve of the sensor was recorded during the three cycles (Figure 7b). It was observed that the sensor has excellent repeatability at low concentrations of TMA gas, but the initial resistance of the sensor showed a slight shift at the concentration of 10 ppm gas. Additionally, the long-term stability of the sensor was tested over one month. As shown in Figure 7c, during this period, the sensor’s response to 2 ppm TMA gas did not exhibit significant changes, indicating excellent stability. However, the recovery of the sensor cannot be 100% after analyte removal. Subsequently, we explored the impact of ambient humidity on the sensing capabilities of the sensor. As shown in Figure 7d, the baseline resistance of the sensor (black line) decreases as humidity increases, and so does the response of the sensor (blue line), but the declining trend shows a linear relationship. In contrast to previously documented TMA sensors, the Ti_3_C_2_T_x_ MXene–MoO_3_ sensor presents notable advantages, including the ability to operate at low testing temperatures, specifically at room temperature. Moreover, it showcases heightened sensitivity and remarkably low detection limits, thus positioning it as a promising advancement in the field of gas sensing technology. This sensor surpasses its predecessors by offering superior performance metrics, paving the way for enhanced detection capabilities in various applications (Table 1).

### 3.3. Design of TMA Concentration Alarm Circuit

**Figure 8:**
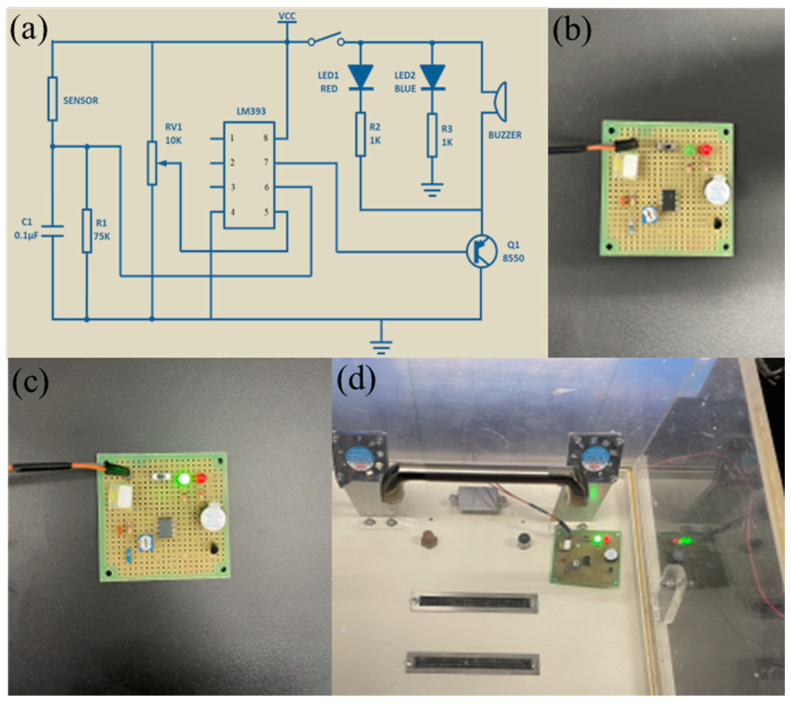
(**a**) TMA concentration over-limit alarm circuit diagram and (**b**) physical object. (**c**) Turn on the alarm circuit switch in the air environment (green light is on). (**d**) Alarm in a 2 ppm trimethylamine environment (red light on).

Additionally, we developed an alarm circuit for the sensor that operates beyond the predefined concentration limits of TMA, serving as a safeguard mechanism. This innovative feature facilitates the generation of an alarm signal, effectively notifying users when the concentration of TMA surpasses a specified threshold. The incorporation of this over-limit alarm circuit enhances the practical utility of the sensor by ensuring timely alerts in situations where TMA concentrations exceed desired or safe levels, thereby contributing to an augmented level of safety and control in various applications. The circuit consists of a Ti_3_C_2_T_x_ MXene–MoO_3_ sensor, voltage comparator, alarm LED light, buzzer, switch, adjustable potentiometer, triode, resistors, capacitors, etc. Different alarm limits can be set by adjusting the resistance of the potentiometer. Figure 8a illustrates the circuit schematic of the TMA concentration detection and over-limit alarm device. Figure 8b is the physical picture of the alarm circuit. The size of the circuit is 6 cm × 6 cm, and it is powered using an external 5 V battery. As shown in Figure 8c, after the alarm device switch is turned on, the green LED lights up, indicating that it is working. As the concentration of TMA gas in the tested environment increases, the resistance value of the sensor also changes accordingly. When the concentration reaches 2 ppm, the TMA gas triggers the activation of the red warning indicator; the green light does not go out, indicating that the ambient TMA concentration exceeds the specified limit. This illumination serves as a visual alert, indicating the need for immediate attention or remedial action due to the elevated presence of TMA in the surroundings. This mechanism guarantees swift detection of potentially perilous TMA concentrations, fostering a proactive approach and upholding safety standards in pertinent settings. Facilitating timely recognition of elevated TMA levels empowers preemptive actions to mitigate risks and uphold regulatory safety protocols within the designated environments (Figure 8d).

### 3.4. Mechanism of TMA Gas-Sensing Properties

**Figure 9:**
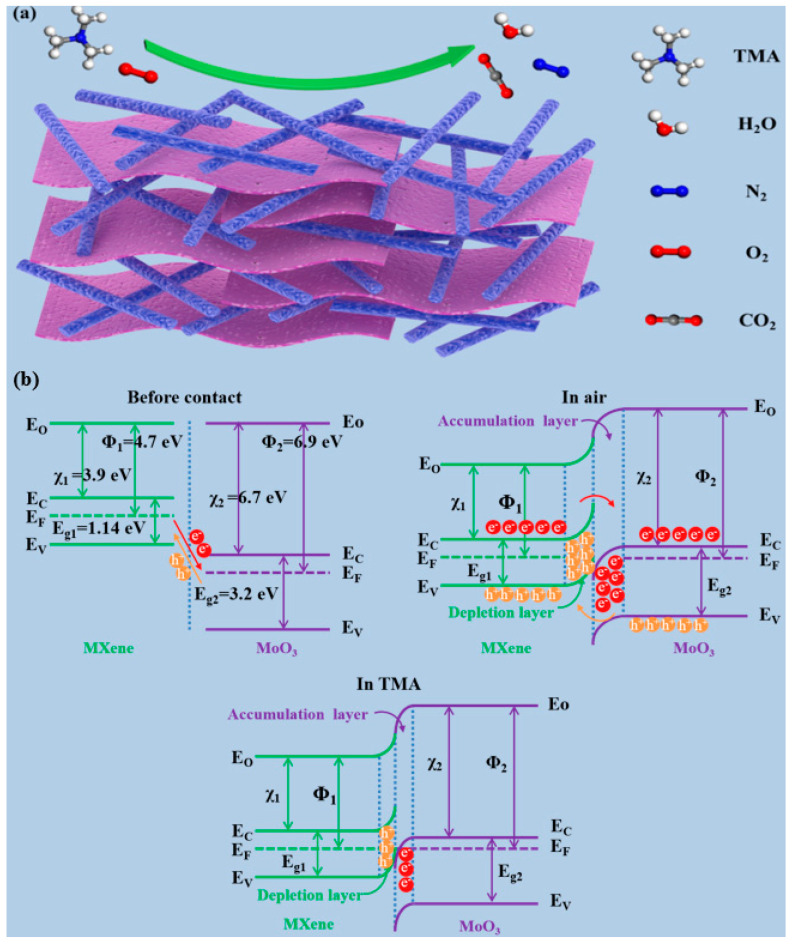
(**a**) Schematic of Ti_3_C_2_T_x_ MXene–MoO_3_ heterostructure in TMA gas. (**b**) Energy band structure diagram of Ti_3_C_2_T_x_ MXene–MoO_3_ heterojunction.

Figure 9 shows the possible sensing mechanism of the Ti_3_C_2_T_x_ MXene–MoO_3_ nanocomposite sensor for TMA gas sensing. Its efficacy hinges on the efficient absorption and desorption characteristics of the sensing substance onto the surface of the MXene–MoO_3_ sensing material, followed by subsequent redox reactions. This effectiveness is contingent upon the ability of the sensing substance to be readily absorbed and released from the surface of the sensing material, thereby facilitating the desired chemical reactions. The dynamic interplay between absorption, desorption, and redox processes dictates the overall performance of the sensor, underscoring the importance of these intricate surface interactions in achieving optimal sensing outcomes. This intricate balance of absorption and desorption dynamics, coupled with redox reactions, governs the sensor’s ability to detect and respond to target stimuli, highlighting the significance of understanding and optimizing these surface phenomena for enhanced sensor performance. Upon exposure to air, the Ti_3_C_2_T_x_ MXene–MoO_3_ nanocomposite-based sensor experiences the adsorption of O_2_ molecules onto the MoO_3_ nanofiber surface. This process results in the entrapment of free electrons within the conduction band of MoO_3_, leading to the formation of O^−^_(ads)_ (Equations (1) and (2)). Consequently, an electron depletion layer manifests on the nanofiber surface. Simultaneously, the transfer of holes occurs from O_2_ gas molecules to the valence band of Ti_3_C_2_T_x_ MXene, resulting in the formation of an augmented layer of hole accumulation on the surface of the p-type Ti_3_C_2_T_x_ MXene.

(1) $$O_{2(\mathrm{gas})}\to O_{2(\mathrm{ads})},$$

(2) $$O_{2(\mathrm{ads})}+2e^{-}\to {2O^{-}}_{\left(\mathrm{ads}\right)},$$

and

(3) $$2{{(\mathrm{CH}}_{3})}_{3}N+21{O^{-}}_{\left(\mathrm{ads}\right)}\to N_{2}{+6\mathrm{CO}}_{2}{+9H}_{2}{O+21e}^{-}$$

When TMA gas molecules are adsorbed, they will consume the pre-adsorbed oxygen according to Equation (3) and release the trapped electrons back to the Ti_3_C_2_T_x_ MXene–MoO_3_ nanocomposite, thereby reducing the resistance value of the sensor.

Additionally, upon the convergence of p-type and n-type semiconductors, the intermingling of holes and electrons at the interface instigates the emergence of heterojunctions, representing a pivotal catalyst in amplifying the sensing proficiency of composite materials. As shown in Figure 9b, the work function of the p-type semiconductor material MXene is 4.7 eV, and the band gap is 1.14 eV, while the work function of the n-type semiconductor MoO_3_ is 6.9 eV, and the band gap is 3.2 eV. It can be seen that the Fermi level of MXene is higher than that of MoO_3_. Therefore, when the two are in contact with each other in the air, electrons will transfer from MXene with a high Fermi level to MoO_3_, and the holes will move in opposite directions until the Fermi level of the two reaches equilibrium. At this time, an electron depletion layer is formed on the MXene side, an electron stacking layer is formed on the MoO_3_ side, and the p–n heterojunction is also formed. When the composite material is placed in a TMA gas environment, the adsorbed oxygen reaction of the trimethylamine and the surface of the material releases electrons to the conduction band of the material, thereby enhancing the conductivity of the sensing material and decreasing the sensor resistance. As a consequence of TMA adsorption and desorption, a potential blockade arises at the interface of Ti_3_C_2_T_x_ MXene and MoO_3_, consequently augmenting the quantity of adsorbed oxygen species. In a TMA environment, the Ti_3_C_2_T_x_ MXene–MoO_3_ hybrid composite sensor undergoes a reaction with the TMA gas and the surface-bound oxygen, liberating electrons and consequently causing a reduction in the thickness of the depletion layer, thereby amplifying the sensor’s responsiveness. Additionally, the unique structures of one-dimensional nanofibers and two-dimensional layered composite materials endow them with higher electron transfer efficiency and larger specific surface areas. By capitalizing on these benefits, the sensor gains access to an increased number of reaction sites, consequently augmenting its sensing capabilities. This strategic utilization of its advantageous features serves to amplify the sensor’s performance in detecting and responding to target stimuli. By leveraging these inherent strengths, the sensor effectively maximizes its potential for accurate and reliable sensing across various applications. This optimized utilization of resources and functionalities underscores the sensor’s capacity to deliver enhanced performance and efficacy in sensing.

## 4. Conclusions

In summary, we have proposed a Ti_3_C_2_T_x_ MXene–MoO_3_ gas sensor and demonstrated its excellent sensing performance toward TMA gas at room temperature. Furthermore, a possible sensing mechanism for this sensor has been proposed. MoO_3_ nanofibers were prepared through electrospinning, with a diameter of about 500 nm, and layered Ti_3_C_2_T_x_ MXene nanosheets were prepared using a classic etching methodTi_3_C_2_T_x_ MXene–MoO_3_ nanocomposites have extremely low detection limits for trimethylamine gas, excellent selectivity, and extremely fast response recovery characteristics (10 s/7 s). The remarkable sensing prowess of the Ti_3_C_2_T_x_ MXene–MoO_3_ sensor in detecting trimethylamine gas is predominantly due to the distinctive morphological characteristics characterized using one-dimensional and two-dimensional structures coupled with the establishment of a p–n heterojunction. In addition, we designed a trimethylamine gas concentration alarm system based on Ti_3_C_2_T_x_ MXene–MoO_3_ sensor and achieved good application effects. Moreover, a potential sensing mechanism has been suggested for this sensor. This proposed mechanism offers insight into the underlying processes through which the sensor detects and responds to the target gases. By delineating the possible pathways and interactions involved in gas sensing, this theoretical framework aids in elucidating the sensor’s operational principles and enhancing our understanding of its functionality. TMA sensors can detect TMA gas in spoiled seafood and exhaled breath with ultrahigh sensitivity and selectivity. It has potential applications in human health monitoring, food safety, and environmental assessment.

## Figures and Tables

**Table 1:** Comparison of TMA sensing performances of the sensor in this work with previously reported sensors.

Materials	Fabrication Method	Conc. (ppm)	Tem. (°C)	Response	LOD (ppm)	Ref.
Pd-ZnO	Solvothermal	1	300	2.9	1	[33]
V_2_O_5_	Hydrothermal	100	240	2.8	10	[34]
V_2_O_3_-Cu_2_O	Electron beam evaporation	3	RT	1.08	3	[35]
Co_3_O_4_/SnO_2_	Hydrothermal	5	175	9.3	5	[36]
MoO_3_/Bi_2_Mo_3_O_12_	Sol-gel	10	170	7.2	0.1	[37]
Rh/ZnO	Thermal evaporation	10	180	11.3	0.055	[38]
MoS_2_/SnO_2_	Hydrothermal	5	230	6	~	[39]
Ti_3_C_2_T_x_ MXene–MoO_3_	Electrospinning- hydrothermal	10	RT	21.2	0.5	This work

## Data Availability

The data that has been used is confidential.
